# Highly Pathogenic Avian Influenza Virus (H5N1) in Frozen Duck Carcasses, Germany, 2007

**DOI:** 10.3201/eid1502.080949

**Published:** 2009-02

**Authors:** Timm C. Harder, Jürgen Teuffert, Elke Starick, Jörn Gethmann, Christian Grund, Sasan Fereidouni, Markus Durban, Karl-Heinz Bogner, Antonie Neubauer-Juric, Reinhard Repper, Andreas Hlinak, Andreas Engelhardt, Axel Nöckler, Krzysztof Smietanka, Zenon Minta, Matthias Kramer, Anja Globig, Thomas C. Mettenleiter, Franz J. Conraths, Martin Beer

**Affiliations:** Friedrich-Loeffler-Institut, Isle of Riems, Germany (T.C. Harder, E. Starick, C. Grund, S. Fereidouni, M. Durban, A. Globig, T.C. Mettenleiter, M. Beer); Friedrich-Loeffler-Institut, Wusterhausen, Germany (J. Teuffert, J. Gethmann, M. Kramer, F.J. Conraths); Bavarian Agency for Health and Food Safety, Erlangen, Germany (K.H. Bogner; R. Repper); Bavarian Agency for Health and Food Safety, Oberschleissheim, Germany (A. Neubauer-Juric); Brandenburg State Central Laboratory, Frankfurt/Oder, Germany (A. Hlinak, A. Engelhardt); Agency for Consumer Protection and Food Safety of Lower Saxony, Oldenburg, Germany (A. Nöckler); National Veterinary Research Institute, Pulawy, Poland (K. Smietanka, Z. Minta)

**Keywords:** Highly pathogenic avian influenza, food safety, H5N1, epidemiology, phylogeny, research

## Abstract

We conducted phylogenetic and epidemiologic analyses to determine sources of outbreaks of highly pathogenic avian influenza virus (HPAIV), subtype H5N1, in poultry holdings in 2007 in Germany, and a suspected incursion of HPAIV into the food chain through contaminated deep-frozen duck carcasses. In summer 2007, HPAIV (H5N1) outbreaks in 3 poultry holdings in Germany were temporally, spatially, and phylogenetically linked to outbreaks in wild aquatic birds. Detection of HPAIV (H5N1) in frozen duck carcass samples of retained slaughter batches of 1 farm indicated that silent infection had occurred for some time before the incidental detection. Phylogenetic analysis established a direct epidemiologic link between HPAIV isolated from duck meat and strains isolated from 3 further outbreaks in December 2007 in backyard chickens that had access to uncooked offal from commercial deep-frozen duck carcasses. Measures that will prevent such undetected introduction of HPAIV (H5N1) into the food chain are urgently required.

## Abstract 2

Article summary line: Phylogenetic and epidemiologic evidence shows incursion of HPAIV into the food chain.

Highly pathogenic avian influenza (HPAI) causes a substantial proportion of deaths in susceptible poultry species, which potentially lead to severe economic losses (*1*). Some HPAI viruses (HPAIV), in particular HPAIV of subtype H5N1 of Asian origin (*2*), exhibit a zoonotic potential, causing rare, but often fatal disease, in humans.

Since this virus was first detected in southern People’s Republic of China in 1996, descendants of this virus have spread among poultry in 3 continents; as yet, only the Americas and Australia have been avoided. In an unprecedented scenario, wild birds have also been widely affected by HPAIV (H5N1) and are believed to have contributed to its transcontinental spread (*3*). In central Europe, HPAIV (H5N1) infections were encountered for the first time in winter 2005–06 and spring 2006, when mainly wild birds, but also a few isolated poultry holdings, were affected. Since August 2006, the virus seemed to have disappeared, but it reemerged in January and February 2007 in Hungary and the United Kingdom (*4*). All of these outbreaks were attributable to virus strains of phyloclusters 2.2, groups A and B, which originated from unknown sources but had phylogenetic links to viruses isolated during outbreaks of HPAI among wild birds at Lake Qinghai in northwestern China in 2005 (*4**,**5**–**8*).

In July and August 2007, introduction of yet another Qinghai-like subcluster of subtype H5N1 viruses, designated 2.2 group C, led to additional cases, first in poultry in the Czech Republic and later in wild birds in France and Germany. In Germany, outbreaks also occurred in several poultry holdings, including 2 large duck-fattening farms. However, the infected ducks did not show clinical symptoms indicative of avian influenza, and no overt excess daily deaths were observed in these holdings. Outbreaks in poultry were spatially and temporally linked to cases in wild birds. Control measures included the culling of >750,000 animals (*9*). These measures seemed to contain the outbreaks because no more cases became apparent after August 2007. Phylogenetic studies indicated that these outbreaks had a common, as yet unidentified, source (*7*).

In December 2007, HPAIV (H5N1) was detected in 3 isolated backyard holdings in the Federal State of Brandenburg in northeastern Germany, although no concomitant cases of subtype H5N1 infection had been detected in wild birds or in poultry since August 2007. This puzzling situation prompted detailed field epidemiologic investigations. These investigations, corroborated by results from wild bird monitoring and from phylogenetic analysis of the respective viruses, indicate that wild birds can be ruled out with high reliability as a source of infection in these cases. Instead, infected duck meat, possibly originating from the German duck-fattening farms affected by the outbreaks in August 2007, might have caused these cases.

## Materials and Methods

### Detection of Virus

RNA from swab samples or tissues was isolated by manual (Viral RNA Kit; QIAGEN, Hilden, Germany, and Tecan Evo 3000 System; Macherey-Nagel, Düren, Germany) procedures. One-step real-time reverse transcription–PCR (rRT-PCR), which specifically amplified fragments of the avian influenza virus (AIV) M, H5, H7, or N1 genes, was performed as reported in European Commission (EC) decision 2006/437/EC. Pathogenicity assessments were based on molecular analysis of the H5 cleavage site by either rRT-PCR (*10*) or sequencing (*11*). Virus isolation was performed in the amnioallantoic cavity of 9- to 11-day-old embryonated hens’ eggs.

### Nucleotide Sequencing of Virus Genes

RNA was prepared from allantoic fluid of inoculated embryonated hens’ eggs. Generation of full-length gene amplification products of the hemagglutinin (HA) gene and sequencing were performed as previously described (*7*). Sequences are publicly available from GenBank: R1359/07-AM 749 443; R1349/07-AM 749 442; R1393/07-AM 773 724; R1400, 1406, 1772, 1779, 1797, 2048/07-AM 914 004/012/014/016/021/026; R3234, 3272, 3294/07-FM 177 119/127/135; and R3248, 3249/07-FM 163 440/448.

### Phylogenetic Analyses

Sequences of full-length HA genes were aligned by using the multiple sequence comparison by log-expectation (MUSCLE) method (www.ebi.ac.uk/Tools/muscle/index.html) and were then subjected to distance matrix calculations (FastME; *12*). Minimal-evolution (ME) trees were built by using the default options of FastME (balanced greedy minimal evolution to build the initial tree and balanced nearest-neighbor interchanges for swapping and optimization). In addition, maximum-likelihood (ML) analysis (TreePuzzle; *13*) was performed by using the public Phylemon server (http://phylemon.bioinfo.cipf.es/cgi-bin/tools.cgi). One thousand (ME) or 200 (ML) bootstrapping cycles were performed.

### Serologic Testing

Poultry serum specimens from affected and in-contact holdings were screened by using commercially available competitive ELISA (cEIA) kits that detect antibodies specific for the nucleocapsid protein (Pourquier AI A Blocking ELISA; Institut Pourquier, Montpellier, France, or ID Screen Influenza A NP Antibody Competition ELISA; ID VET, Montpellier, France) according to the manufacturers’ instructions. Internal validation data showed that performance characteristics of these 2 assays were largely comparable with those of duck sera. Consequently, they were used interchangeably in the different laboratories. Positive serum specimens were further analyzed by hemagglutination inhibition (HI) assay according to 2006/437/EC. Antigens prepared from subtypes H5N2 (A/ostrich/Denmark/72420/96; Veterinary Laboratories Agency [VLA], Weybridge, UK), H5N1 (NIBRG14, NIBSc, UK), and H7N7 (A/tk/England/647/77; VLA) viruses were used.

## Results

### Outbreak Detection and Field Epidemiologic Investigations

#### Bavaria

In an industrial duck-fattening farm (farm A) in Bavaria, Germany, a slight increase of daily mortality rates ranging from 0.7% to 1.8% from August 19, 2007, onward was registered in barn A/15, which prompted swab sampling on August 22, 2007 (Table). Although initially *Riemerella* spp. were detected, differential diagnostic measures included PCRs for AIV (H5N1), which yielded positive results. HPAIV (H5N1) was finally confirmed on August 25, 2007, which led to the culling of all 170,000 ducks kept at that time at farm A. Further sampling at culling led to detection of HPAIV (H5N1) in 3 other barns of farm A (nos. 10, 12, and 13; Table).

**Table:** Summary of investigations for HPAIV (H5N1) infections in industrial duck-fattening farms A, B, and C by rRT-PCR, sequencing and serologic analyses, Germany, 2007*

Farm/barn-unit	Date of housing	Herd size	Date of culling	Cumulative proportion of deaths, %	Duckling age, d	Swab samples†	Serum samples‡	Tissue samples†
A/10	13 Jul	12,015	25 Aug	14.4§	43	50/6 (25 Aug)	30/30/6	–
A/12	16 Jul	39,165	26 Aug	12.5	41	50/39 (25 Aug)	30/13/1	–
A/13	13 Jul	14,000	25 Aug	14.4§	43	50/23 (25 Aug)	30/30/4	–
A/15	1 Aug	45,696	25 Aug	10.2	25	25/13 (22 Aug)	–	–
B/1-1	29 Jun	32,540	11 Aug¶	20.3 (Figure 1, panel C)	43	–	–	7/(1)
B/1-1	14 Aug	35,175	9 Aug	ND	24	519/0 (5 Sep)	106/5/0	–
B/1-2	17 Aug	35,000	9 Aug	ND	21	511/0 (5 Sep)	109/5/0	–
B/2-4	20 Jul	35,860	9 Sep	5.9 (Figure 1, panel D)	50	511/6# (5 Sep)	126/105/52	122/1
B/3-5	25 Apr	22,550	14 Jun¶	12.6 (Figure 1, panel A)	50	–	–	–
B/3-5	20 Jun	36,300	8 Jan¶	8.3 (Figure 1, panel B)	42	–	–	34/2
B/3-5	7 Aug	35,860	9 Oct	2.4	34	515/0 (5 Sep)	125/3/0	–
B/3-6	10 Aug	34,650	9 Oct	1.2	31	519/0 (5 Sep)	130/1/0	–
C	19 Jul	28,000	9 Oct	2.2	52	515/0 (5 Sep)	91/3/0	–

*HPAIV, highly pathogenic avian influenza; rRT-PCR, real-time reverse transcription–PCR; ND, no data available. †Total number of samples (oropharyngeal swabs; lung and central nervous system tissues) examined by rRT-PCR and sequencing/no. of subtype H5N1-positive samples; date of swab collection is indicated in parentheses. ‡Total number of serum samples examined/nos. positive for nucleocapsid protein–specific antibodies/nos. positive in H5-specific hemagglutination inhibition assay (titer ≥16). §Combined data on proportion of deaths for barns A/10 and A/13; increased losses in barn A/10 occurred after early August 2007. ¶Date of regular slaughter. HPAIV (H5N1) was found in retained frozen duck carcasses of flocks B/3–5 (hatched 6/20/07) and subtype H5N1–specific RNA was present in 1 frozen duck carcass of flock B/1–1 (hatched 6/29/07), but the sample could not be pathotyped due to low viral genome loads. #Due to low viral loads the H5N1 pathotype could be confirmed by sequencing a hemaglutinin fragment in only 2 cases.

**Figure 1:**
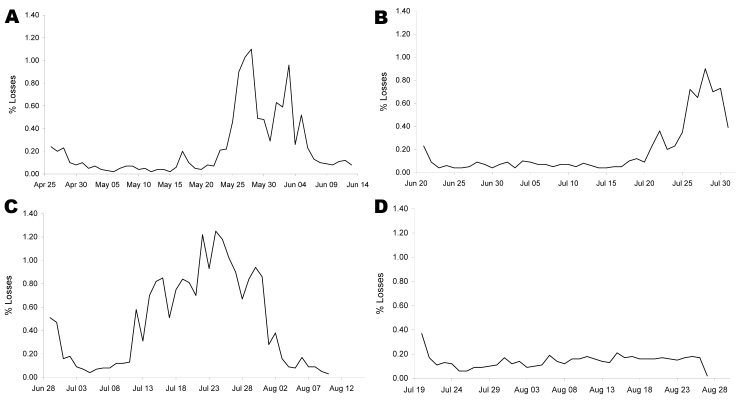
Deaths of ducks in different barn units of farm B, Bavaria. A) Barn B/3–5; duck hatched April 25, 2007, slaughtered June 14, 2007, at an abattoir in Lower Saxony: no material available for testing. B) Barn B/3–5; duck hatched June 20, 2007, slaughtered August 1, 2007, at farm A): 2 highly pathogenic avian influenza virus (HPAIV) A (H5N1)–positive samples detected in retained samples of frozen duck carcasses; virologic and serologic results suggestive of a recent HPAIV (H5N1) infection are listed in the Table. C) Barn B/1–1; duck hatched June 29, 2007, slaughtered August 11, 2007, at farm A; subtype H5N1–specific RNA was detected in 1 retained sample of a frozen duck carcass, but the pathotype could not be determined due to low viral loads. D) Barn B/2–4; duck hatched July 20, 2007, culled September 9, 2007; virologic and serologic results suggestive of a recent HPAIV (H5N1) infection are listed in the Table.

Farm A also operated a large regional poultry abattoir. Thus, a considerable number of contact farms, most of them keeping ducks for fattening, including farms B and C, were identified. Except for farms B and C, no clinical, virologic, or serologic evidence for spread of virus was obtained in monitoring investigations. Farms B and C were serviced by the same crew of poultry workers and, hence, were treated as a single epidemiologic unit. No clinical evidence for an HPAIV infection was obtained on August 28, 2007, and an initial virologic investigation of 60 oropharyngeal and cloacal swabs yielded negative results. However, residual lung tissues obtained from 2 retained frozen carcasses of ducks that had been reared at farm B (barn B/3–5) and slaughtered at the abattoir at farm A on August 1, 2008, tested positive for HPAIV (H5N1) (Table). In this fattening flock, a slightly increased cumulative proportion of deaths (8.3%) was evident (Figure 1, panel B). After these findings, swab sampling was increased to 450 per barn unit at farms B and C to ensure detection of HPAIV infection at a prevalence of 1% with 99% confidence. In addition, serologic surveillance was initiated. No evidence for any infection by AIV H5 was found at farm C (Table). Farm B, however, housed at least 1 flock of ducks ready for slaughter and marketing (Table: B/2–4) that showed serologic evidence for widespread infection with AIV H5. In 4 oropharyngeal swabs of this flock, low genome loads of AIV (H5N1) were detected; 2 swabs yielded sufficient material to confirm, by sequencing, the presence of HPAIV. The low prevalence of active viral infection contrasted the high H5-specific seroprevalence, which indicated that the peak of infection in this flock had passed probably 2–3 weeks before swabbing for virologic testing had been initiated on September 5, 2007 (Table). The overall cumulative losses in this flock nevertheless amounted to only 5.9% (Table; Figure 1, panel D). The culling of poultry on farms B and C was completed on September 10, 2007, and all poultry of farms B and C slaughtered after July 31, 2007, at the abattoir at farm A were confiscated and destroyed.

Retrospective analysis of duck deaths at farm B showed modestly enhanced cumulative values in at least 3 fattening flocks during midterm or toward the end of the fattening period (Table; Figure 1, panels A–C). Virologically, HPAIV (H5N1)–positive animals were detected retrospectively in barn B/3–5, which had hatched on June 20, 2007, and showed a cumulative proportion of deaths of 8.3% (Table; Figure 1, panel B). Inconclusive results were obtained for the flock that had hatched on June 29, 2007 (Table; Figure 1, panel C). Another suspected fattening flock (hatching date April 25, 2007; Table; Figure 1, panel A) could not be retrospectively analyzed. In none of these flocks did daily mortality rates exceed 2%, the legal cut-off for mandatory targeted etiologic investigations including for avian influenza viruses. However, cumulative proportion of deaths amounted to up to 20.3%.

#### Brandenburg

On December 10, 2007, three of 11 chickens were found dead at a backyard holding in the Federal State of Brandenburg in northeastern Germany. Another chicken had died on December 7, and a reduction in egg production on the farm was observed. HPAIV (H5N1) was detected in 2 birds submitted for pathologic and laboratory testing on December 11. Clinical signs in the chickens that were still alive on December 12 and 13 included lethargy, ruffled feathers, reduced mobility, and cyanosis of the combs and wattles. Two of these birds died on December 12, and another 3 died on December 13. The remaining chicken was culled on December 14 after HPAIV (H5N1) had been confirmed in samples submitted to the national reference laboratory on that day. Two more cases of HPAIV (H5N1) infection in similar backyard chicken holdings were detected on December 20 and 23, 2007, respectively, in the same region but separated by 80–120 km.

No direct connections between these holdings were identified. As judged from the lack of recent movements of animals, vehicle traffic, and contacts of owners, no hints toward an incursion or further spread of virus was evident through these routes. Holdings were situated in areas rich in migratory birds. In addition, only 1 further condition appeared to be shared between these holdings: Within 2 to 4 days before the outbreaks, the chickens had access to raw offal of deep-frozen duck carcasses that had been purchased from a supermarket chain in October in that region. These ducks had been frozen and were consumed just 3–5 days before the outbreaks. Owners of the third holding refused to make any specific comments concerning this point, but circumstantial evidence points toward a similar scenario. At the time of investigation, no further material from any of these deep-frozen duck carcasses was available for analysis.

### Outbreak-associated Surveillance Activities

#### Bavaria

From January 1, 2007, until August 31, 2007, a total of 1,236 wild birds were tested in Bavaria for avian influenza viruses. From June 24 through August 3, 2007, HPAIV (H5N1) was detected in 19 aquatic birds (mute swans, gray lag and Canada geese, tufted ducks). Extensive serologic (2,107 samples) and virologic (5,833 samples) surveillance in altogether 46 further contact holdings to farm A extending over the whole area of Germany did not yield any indication of past or ongoing AIV H5 infections.

#### Brandenburg

In 2007, a total of 1,696 wild birds were tested in Brandenburg for avian influenza virus. In December 2007 and in January 2008, 283 and 162 wild birds, respectively, were tested with negative results. HPAIV (H5N1) was not detected in any of the samples. Serologic testing of 4,040 blood samples and virologic testing of 2,836 swab samples from poultry had negative results for subtype H5N1 in 2007.

### Phylogenetic Analyses

The HA gene of 1 representative virus isolate of each of the 3 Brandenburg holdings and of the virus isolated from duck meat were sequenced. Sequence comparisons showed a very close relationship between viruses from the 3 Brandenburg holdings and from the Bavarian duck meat. Within the HA gene, complete identity was found between 2 viruses in the Brandenburg holdings and the virus in duck meat; the third Brandenburg virus was distinguished by a single nonsynonymous mutation (K65R).

**Figure 2:**
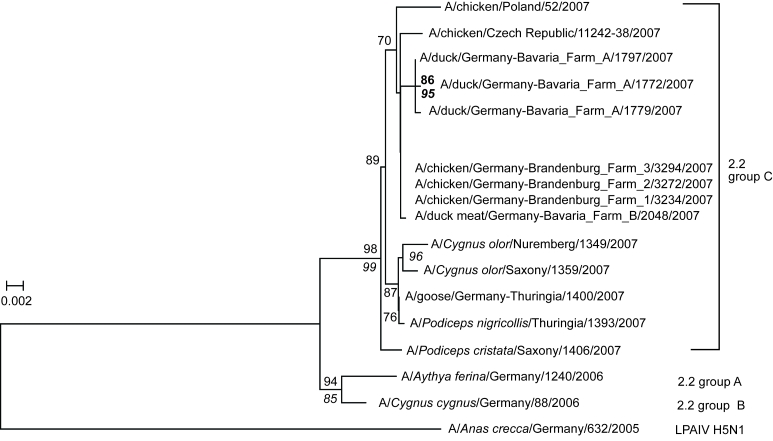
Phylogetic tree of the hemagglutinin (HA) gene (full-length sequence) of highly pathogenic avian influenza virus (HPAIV) (H5N1) detected in poultry from Brandenburg and Bavaria, Germany, in 2007, including sequences of wild birds and poultry from neighboring countries. Sequence of the Czech poultry isolate is supported by GenBank. The tree was constructed by using a minimal-evolution algorithm; numbers represent bootstrap values after 1,000 replications. A maximum-likelihood (ML)–based tree resulted in a similar topology; italicized numbers indicate bootstrap values of the ML tree after 200 replications. Scale bar indicates substitutions per site. The HA sequence of A/chicken/Czech Republic/11242–38/2007 (H5N1) was extracted from GenBank accession no. EU 443553. LPAIV, low pathogenicity avian influenza virus.

Phylogenetic analysis of the full-length HA gene of these and other HPAIV (H5N1) viruses isolated in 2007 in Germany and neighboring countries is presented in Figure 2. All viruses belonged to cluster 2.2, group C. This lineage had not been detected during the 2006 outbreaks among wild birds in Germany and, therefore, most likely represents a new incursion in 2007 (*7*). Viruses from the Bavarian holding A clustered separately from those of the Bavarian holding B. Brandenburg viruses A–C clustered with the virus sequences from duck meat originating from Bavarian holding B (Figure 2).

## Discussion

The high homology of RNA sequences of the HA genes derived from viruses that caused outbreaks at 3 different holdings in the German Federal State of Brandenburg and virus isolated from deep-frozen duck carcasses (A/duck meat/Bavaria/2048/2007) clearly points to a close epidemiologic link between these outbreaks. According to theoretical consideration regarding the mutation rate of influenza A viruses (*14*) and to practical experiences during outbreaks (*4*), if HPAIV had gone through a transmission chain consisting of several hosts, more extensive sequence differences would likely have resulted. This likelihood is further emphasized by the comparison of 3 subtype H5N1 virus isolates derived from a single barn of farm A (Figure 2); these viruses exhibit greater variability among each other than do viruses from the 3 different affected backyard holdings in Brandenburg. Also, distinct sequences of viruses were detected from poultry in Poland, where outbreaks occurred at the same time as the Brandenburg cases, which renders an incursion from this source highly unlikely. Among the Brandenburg cases, no epidemiologic links could be detected except that, as proven in 2 cases and assumed to have occurred in the third case, backyard chickens had access to uncooked offal from duck meat purchased separately in different supermarkets of the same national chain.

Oral uptake of virus is an efficient way of transmitting HPAIV among poultry and mammals (*15**,**16*). Infectious virus in titers of up to 10^7.2^ 50% egg infectious doses per gram in muscles of infected chickens, ducks, and quails has been repeatedly demonstrated (*17**,**18*). Although feeding of poultry offal to poultry or livestock is legally prohibited in Germany, unintended access of backyard poultry to poultry meat and organs is sometimes possible. If such offal is contaminated with HPAIV, transmission becomes possible, and isolated outbreaks like those reported from Brandenburg may ensue. However, this circumstance would require the presence of HPAIV in meat destined for human consumption. This possibility had previously been estimated to be low (*19*).

Unfortunately, no experimental evidence could be produced that unequivocally links the Bavarian farm B and Brandenburg backyard outbreaks because no material was left for virologic examination from the suspected deep-frozen duck carcasses. Therefore, we tried to collect further epidemiologic data by fully retracing the origin of the frozen ducks purchased by the owners of the backyard chickens in Brandenburg. This attempt included authorities in the involved Federal States and the management of the supermarket chain that had sold the duck meat.

**Figure 3:**
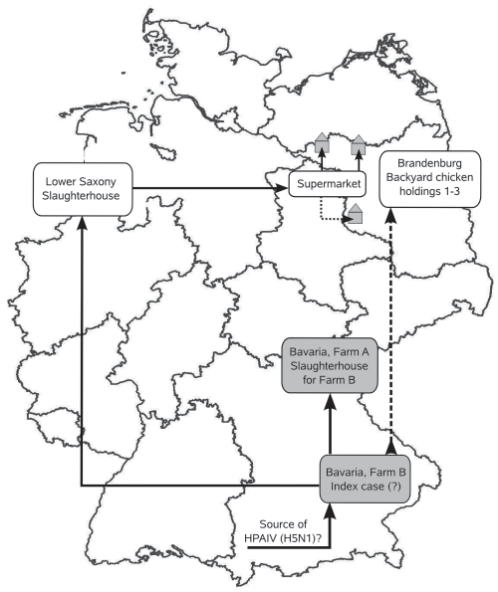
Possible pathway of transmission of highly pathogenic avian influenza virus (HPAIV) (H5N1) from farm B, Bavaria, to 3 backyard chicken holdings in Brandenburg (gray house symbols) based on phylogenetic and circumstantial epidemiologic evidence. Viruses of these cases were virtually identical, although they were separated by 4 months (August and December, 2007) and ≈400 km without linking outbreaks. In contrast, other viruses occurring at the same time (August) in Bavaria in wild birds or in farm A were distinguishable from those of farm B. The same was true for viruses detected in Poland (close to Brandenburg) in December. Therefore, a direct epidemiologic link between farm B and the outbreaks in Brandenburg was suspected (dashed arrow). From June 14 through June 22, 2007, three fattening flocks from farm B were slaughtered in Lower Saxony (angled arrow). These included flock B/3-5 with elevated proportion of deaths (Figure 1, panel A). Slaughtering lots 724/725, which contained ducks from farm B flocks with an elevated cumulative proportion of deaths, were distributed in Brandenburg supermarkets (horizontal arrow). Frozen duck carcasses from these lots had been purchased by the owners of the Brandenburg chicken holdings, and in 2 of the 3 outbreaks, owners admitted that chickens had access to uncooked offal from these carcasses before the outbreaks.

The duck meat sold in autumn 2007 by the supermarket chain in Brandenburg and in the neighboring Federal States of Berlin and Mecklenburg–Western Pomerania had been purchased from a slaughterhouse in the Federal State of Lower Saxony (lots 724, 725; Figure 3). Direct links between Bavarian farm B and the slaughterhouse in Lower Saxony existed because slaughtering lots 724 and 725 included ducks from 3 fattening flocks from farm B. These ducks were slaughtered on June 14, 19, and 22, 2007, in the abattoir in Lower Saxony, because the most frequently used abattoir in Bavaria at farm A was closed for holidays (Figure 3). Among the 3 flocks was the one from barn B/3–5, which showed a suspiciously elevated cumulative proportion of deaths of 12.6% (Table; Figure 1, panel A).

This circumstantial evidence points toward a transient and limited incursion of HPAIV (H5N1) into duck meat destined for human consumption. Therefore, enhanced virologic screening of fattening ducks has been initiated in Germany. Also, reporting obligations related to deaths in duck flocks were adopted. Since the end of the Brandenburg outbreaks, a single, unrelated recurrence of HPAIV (H5N1) in Germany was detected in poultry in 2008, but none has been detected in wild birds. To date, no clinical cases of human infection with subtype H5N1 have been reported in Germany.

In avian hosts, the clinical picture of an HPAIV infection depends, among other factors, on the species affected (*20*). In particular, domestic waterfowl showed substantial variations in clinical features resulting from infection with strains of HPAIV (H5N1) of Asian origin (*21*). Factors influencing the clinical course relate to species, age of animals, and the virus strain (*22*,*23*). The cited experimental data as well as reported evidence from the field (*24*) show that an introduction and subsequent spread of HPAIV (H5N1) in duck flocks is likely clinically silent. Also in the cases reported here, hardly any clinical symptoms suggestive of an HPAIV infection, in particular, neurologic manifestations, were evident. Increased proportion of deaths, as seen in some of the described duck-fattening flocks, might have been fueled by bacterial co-infections, e.g., by those caused by *Riemerella* spp. Through silently but productively infected ducks, an endemic status of HPAIV (H5N1) infection can be established and perpetuated (*25*). Strains isolated from such endemic infections induce no clinical symptoms in ducks but retain high pathogenicity for chickens and turkeys. No sign of even widespread infection would be clinically apparent until the virus has become more established in flocks of highly vulnerable (gallinaceous) species (*26*). Such mechanisms are obviously not restricted to subtropical Southeast Asia.

In conclusion, our data show that incursions of recent strains of descendants of Qinghai lineage HPAIV (H5N1) strains into industrial duck-fattening holdings in Europe may be clinically silent, even in young ducklings. If daily mortality rates remain low, an increase in cumulative mortality may still be evident and should prompt specific virologic investigations. Undetected HPAIV (H5N1) infections of domestic waterfowl destined for human consumption raises the risk for human infection when infected birds or contaminated meat products are handled. Thus, measures must be strengthened that can prevent this zooanthroponotic virus from entering the food chain through contaminated duck meat products and spreading further. Intensified monitoring of duck herds for HPAIV infection that does not rely on syndromic surveillance would be required first. In the outbreaks reported here, the power of serologic assays to detect virus incursions is notable, compared with results by rRT-PCR. Species-independent cEIA assays detecting antibodies specific for the nucleocapsid protein of influenza A viruses were more sensitive than HI assays that used 2 different H5 antigens. Despite the intrinsically higher sensitivity of most ELISAs, the discrepancy in this study might have been aggravated by not using antigen of the outbreak viruses in HI assays. Nevertheless, cEIAs would be suitable for high-throughput analysis in extensive monitoring programs while HI techniques would not.

## References

[1] Swayne DE, Suarez DL. Highly pathogenic avian influenza. Rev Sci Tech. 2000;19:463–82. 10935274 10.20506/rst.19.2.1230

[2] Capua I, Alexander DJ. Animal and human health implications of avian influenza infections. Biosci Rep. 2007;27:359–72. 10.1007/s10540-007-9057-9 17597393

[3] Sims LD, Domenech J, Benigno C, Kahn S, Kamata A, Lubroth J, Origin and evolution of highly pathogenic H5N1 avian influenza in Asia. Vet Rec. 2005;157:159–64. 16085721 10.1136/vr.157.6.159

[4] Irvine RM, Banks J, Londt BZ, Lister SA, Manvell RJ, Outtrim L, Outbreak of highly pathogenic avian influenza caused by Asian lineage H5N1 virus in turkeys in Great Britain in January 2007. Vet Rec. 2007;161:100–1. 17694626 10.1136/vr.161.3.100

[5] Chen H, Li Y, Li Z, Shi J, Shinya K, Deng G, Properties and dissemination of H5N1 viruses isolated during an influenza outbreak in migratory waterfowl in western China. J Virol. 2006;80:5976–83. 10.1128/JVI.00110-06 16731936 PMC1472608

[6] Nagy A, Machova J, Hornickova J, Tomci M, Nagl I, Horyna B, Highly pathogenic avian influenza virus subtype H5N1 in mute swans in the Czech Republic. Vet Microbiol. 2007;120:9–16. 10.1016/j.vetmic.2006.10.004 17113249

[7] Starick E, Beer M, Hoffmann B, Staubach C, Werner O, Globig A, Phylogenetic analyses of highly pathogenic avian influenza virus isolates from Germany in 2006 and 2007 suggest at least three separate introductions of H5N1 virus. Vet Microbiol. 2008;128:243–52. 18031958 10.1016/j.vetmic.2007.10.012

[8] Gall-Reculé GL, Briand FX, Schmitz A, Guionie O, Massin P, Jestin V. Double introduction of highly pathogenic H5N1 avian influenza virus into France in early 2006. Avian Pathol. 2008;37:15–23. 10.1080/03079450701774835 18202945

[9] Scheibl P. Field report from large-scale killing of ducks [in German]. Dtsch Tierarztl Wochenschr. 2008;115:158–61. 18500150

[10] Hoffmann B, Harder T, Starick E, Depner K, Werner O, Beer M. Rapid and highly sensitive pathotyping of avian influenza A H5N1 virus by using real-time reverse transcription-PCR. J Clin Microbiol. 2007;45:600–3. 10.1128/JCM.01681-06 17182758 PMC1829000

[11] Commission decision 2006/437/EC of 4 August 2006 approving a diagnostic manual for avian influenza as provided for in council directive 2005/94/ECEU decision 2006/437/EC [cited 2008 Jan 5]. Available from http://eur-lex.europa.eu/LexUriServ/LexUriServ.do?uri=OJ:L:2006:237:0001:0027:EN:PDF

[12] Desper R, Gascuel O. Fast and accurate phylogeny reconstruction algorithms based on the minimum-evolution principle. J Comput Biol. 2002;9:687–705. 10.1089/106652702761034136 12487758

[13] Schmidt HA, Strimmer K, Vingron K, von Haeseler A. TREE-PUZZLE: maximum likelihood phylogenetic analysis using quartets and parallel computing. Bioinformatics. 2002;18:502–4. 10.1093/bioinformatics/18.3.502 11934758

[14] Chen R, Holmes EC. Avian influenza virus exhibits rapid evolutionary dynamics. Mol Biol Evol. 2006;23:2336–41. 10.1093/molbev/msl102 16945980

[15] Keawcharoen J, Oraveerakul K, Kuiken T, Fouchier RA, Amonsin A, Payungporn S, Avian influenza H5N1 in tigers and leopards. Emerg Infect Dis. 2004;10:2189–91. 15663858 10.3201/eid1012.040759 PMC3323383

[16] Das A, Spackman E, Thomas C, Swayne DE, Suarez DL. Detection of H5N1 high-pathogenicity avian influenza virus in meat and tracheal samples from experimentally infected chickens. Avian Dis. 2008;52:40–8. 10.1637/8093-082107-Reg 18459294

[17] Antarasena C, Sirimujalin R, Prommuang P, Blacksell SD, Promkuntod N, Prommuang P. Tissue tropism of a Thailand strain of high-pathogenicity avian influenza virus (H5N1) in tissues of naturally infected native chickens (*Gallus gallus*), Japanese quail (*Coturnix coturnix japonica*) and ducks (*Anas* spp.). Avian Pathol. 2006;35:250–3. 10.1080/03079450600714510 16753617

[18] Swayne DE, Beck JR. Experimental study to determine if low-pathogenicity and high-pathogenicity avian influenza viruses can be present in chicken breast and thigh meat following intranasal virus inoculation. Avian Dis. 2005;49:81–5. 10.1637/7260-081104R 15839417

[19] Greiner M, Müller-Graf C, Hiller P, Schrader C, Gervelmeyer A, Ellerbroek L, Expert opinion based modelling of the risk of human infection with H5N1 through the consumption of poultry meat in Germany. Berl Munch Tierarztl Wochenschr. 2007;120:98–107. 17416131

[20] Alexander DJ. A review of avian influenza in different bird species. Vet Microbiol. 2000;74:3–13. 10.1016/S0378-1135(00)00160-7 10799774

[21] Sturm-Ramirez KM, Hulse-Post DJ, Govorkova EA, Humberd J, Seiler P, Puthavathana P, Are ducks contributing to the endemicity of highly pathogenic H5N1 influenza virus in Asia? J Virol. 2005;79:11269–79. 10.1128/JVI.79.17.11269-11279.2005 16103179 PMC1193583

[22] Pantin-Jackwood MJ, Swayne DE. Pathobiology of Asian highly pathogenic avian influenza H5N1 virus infections in ducks. Avian Dis. 2007;51(Suppl):250–9. 10.1637/7710-090606R.1 17494561

[23] Pantin-Jackwood MJ, Suarez DL, Spackman E, Swayne DE. Age at infection affects the pathogenicity of Asian highly pathogenic avian influenza H5N1 viruses in ducks. Virus Res. 2007;130:151–61. 10.1016/j.virusres.2007.06.006 17658647

[24] Smith GJ, Fan XH, Wang J, Li KS, Qin K, Zhang JX, Emergence and predominance of an H5N1 influenza variant in China. Proc Natl Acad Sci U S A. 2006;103:16936–41. 10.1073/pnas.0608157103 17075062 PMC1636557

[25] Songserm T, Jam-on R, Sae-Heng N, Meemak N, Hulse-Post DJ, Sturm-Ramirez KM, Domestic ducks and H5N1 influenza epidemic, Thailand. Emerg Infect Dis. 2006;12:575–81. 16704804 10.3201/eid1204.051614 PMC3294714

[26] Gilbert M, Xiao X, Pfeiffer DU, Epprecht M, Boles S, Czarnecki C, Mapping H5N1 highly pathogenic avian influenza risk in Southeast Asia. Proc Natl Acad Sci U S A. 2008;105:4769–74. 10.1073/pnas.0710581105 18362346 PMC2290786

